# The community pharmacy practice change towards patient-centered care in Saudi Arabia: a qualitative perspective

**DOI:** 10.1186/s40545-020-00267-7

**Published:** 2020-09-14

**Authors:** Muhammad Kamran Rasheed, Abdulmajeed Alqasoumi, Syed Shahzad Hasan, Zaheer-Ud-Din Babar

**Affiliations:** 1 Department of Pharmacy Practice, College of Pharmacy, Qassim University, Buraydah, Kingdom of Saudi Arabia; 2 Department of Pharmacy, University of Huddersfield, Huddersfield, HD1 3DH UK

**Keywords:** Community pharmacy, Saudi Arabia, Qualitative research, Stakeholders, Patient-centered care, Healthcare consumer

## Introduction

The Kingdom of Saudi Arabia (KSA) has made tremendous improvements in its health care infrastructure in a short span of time [1]. Socioeconomic changes in the KSA led to the rise of the middle-class, which means an inevitable increased future demand for health care [2–4]. Although per capita total healthcare has increased to US$1004 in 2015 and the total health expenditure is predicted to increase in the US$44 billion by 2019, growing lifestyle diseases had become a major challenge for the government of the KSA [5–7]. Consequently, there is an increased burden on the Ministry of Health (MoH) to provide primary health care, and the shortages of trained health care professionals at primary health care settings make the situation more challenging [2, 8]. The changing cultural expectation of the need to see a doctor for relatively minor symptoms also increases demand, although arguably not needed [1, 2, 6, 7].

Previous studies in the KSA have reported that many Saudi healthcare consumers frequently visit their local community pharmacy for multiple reasons, including disease-related advice, purchasing over-the-counter (OTC) or cosmetic products, and procuring prescription medicines for the treatment or prevention of the progression of chronic conditions [2, 3, 9–11]. The community pharmacies are highly accessible across the KSA, and it is convenient for health care consumers to approach the community pharmacist without appointments or referrals [3, 10, 12]. Furthermore, Saudi health care consumers expect the community pharmacists to play a significant role in improving the health outcomes [3, 9, 10]. Therefore, pharmacists’ involvement in raising public health awareness at the community level and educating the public on prevention and monitoring of lifestyle diseases and improving the quality use of medicines can play a significant role in decreasing the morbidity and mortality from chronic diseases like ischemic heart diseases and diabetes mellitus [3, 5–7, 13].

The potential for community pharmacists to fill the capacity gap in providing extended patient-centered care services to patients in the KSA is a much-needed initiative [3, 14]. Given that the Saudi government has emphasized public-private partnerships for effective primary health care in its vision 2030, there is a significant opportunity for community pharmacies to be part of that vision [2, 4, 15].

Numerous studies conducted in the KSA agreed in principle about the “content” that means a change in the practice of the community pharmacy from its traditional role of providing only dispensing services to the consumers to the providers of patient-centered care [2, 9, 11, 12, 16, 17]. However, there is a paucity of literature on “context,” which means how the internal and external factors can lead to that change and also the “process” and steps needed for the change [18]. Therefore, this study aimed to identify the views of key stakeholders (individuals representing the government and non-government, consumer health organizations) about the extended role of community pharmacy in primary health care set-up in the KSA. The study also sought to identify drivers, motivators, and facilitators of the change process in community pharmacy towards providers of patient-centered care.

## Methods

A qualitative research approach was used in this study to explore and understand stakeholders’ opinions on extended community pharmacy services in the KSA [19–21].

### Sampling of respondents and inclusion criteria

A purposeful sample of stakeholders in pharmacy practice was selected to participate in the study. Prior knowledge of the education and health sectors of the KSA and recommendations from the study participants were used to identify potential stakeholders. The stakeholders comprised of participants who were either independent community pharmacy owners or chief operating officers of community pharmacies, academics, and researchers in pharmacy practice; high-ranking Ministry of Health officials; and people from professional pharmacy organizations in the KSA. An invitation was sent to twenty stakeholders based on the criteria mentioned above. Fifteen stakeholders agreed to participate in the interview. Those stakeholders include five officials from community pharmacies, one healthcare consultant, six officials from the Ministry of Health, and three academics and researchers in universities (see Table 1 for the description of the sample).

**Table 1:** Overview of sample

*N* = 15	Number of participants (stakeholders)
Ministry of Health (MoH)	
Al-Qassim	3
Riyadh	1
Jeddah	2
Healthcare consultant (HC)	1
Academics and research (AR)	
Al-Qassim	1
Riyadh	2
Community pharmacy (CP)	
Owner	1
CEO	2
Director	2
Gender	
Male	12
Female	3

### Instrument development

For conducting a qualitative interview, a semi-structured interview guide was developed to gather the opinions of the selected participants. The semi-structured interview was preferred to obtain qualitative data because it provides detailed descriptions of interviewee’s experiences. The interview guide was prepared after a literature review [22-24] then discussed and refined by the study investigators (Table 2). The interview guide was then sent to an external evaluator for the feedback (e.g., relevance and accuracy). The final draft of the interview guide was piloted in two interviews at the beginning of this study [19, 23-25]. A study information sheet was developed, which contains information about research aims and objectives, researcher’s background, and benefits of conducting this study. The interview guide and the information sheet were translated into the Arabic language by two native speakers through the forward and backward translation method. The interview guide and the study information sheet were sent to all stakeholders prior to conducting interviews.

**Table 2:** Final interview guide

1. Can you please describe the services community pharmacy currently provides in Saudi Arabia? 2. What are your views about the current role and practices of community pharmacist in Saudi Arabia in providing services to the consumers? 3. In your opinion, how a community pharmacist can help in managing consumer’s medications and diseases? 4. According to your opinion, which patient-centered care services in community pharmacy would be (or are generally) preferred by consumers? 5. In your opinion, how effective patient-centered care services can be implemented in community pharmacies of Saudi Arabia? 6. What type of services would you like to see in community pharmacy providing in the future to better support health care consumers? 7. What issues do you think community pharmacy might face in shifting toward patient-centered care services? 8. What are the barriers/problems associated with implementation of patient-centered care services (existing and future) in community pharmacy practice in Saudi Arabia?	

### Data collection process

Each stakeholder was contacted via email to identify if he/she would be interested in participating in the study. In some instances, individuals were contacted personally to participate in the study. All stakeholders were given an option for conducting interviews in English or Arabic. Twelve stakeholders preferred English, while three interviews were conducted in the Arabic language. For the Arabic interviews, a native speaker of the Arabic language, also fluent in English, accompanied the lead researcher **(MR).** On confirmation of a participant’s verbal and written consent to participate, the study information sheet and interview guide were emailed to them. All interviews were audio-recorded on a good-quality recording device. We applied the learning from one interview to go to the next interview to probe more accurately [19, 22, 23].

During the interview process, our main aim was to discover the interviewee’s framework of meanings, and the research task is to avoid imposing the researcher’s structures and assumptions on the interviewee’s account as far as possibl e[18, 23, 24]. All interviews were interactive and sensitive to the language and concepts, and the interviewer tried to keep the agenda open and flexible. The main aim of the interview is to go below the surface of the topic being discussed, explore what stakeholders say in as much detail as possible, and uncover new ideas or areas that were not anticipated at the outset of the research. The questions of the interviews were open-ended, neutral, sensitive, and clear to the interviewee. The interview was started with easy-to-answer questions and then proceeds to more specific questions that were derived from the interviewee’s answers. Eight of the fifteen interviews had to be conducted by telephone because of logistical and resource constraints and difficulty in finding mutually acceptable time for face-to-face interviews. After 13 interviews, no new information from the interviewees was forthcoming; data saturation was therefore reached, and the decision has been taken to stop after the 15th interview.

### Data analysis

All audio recordings were transcribed verbatim, keeping the exact wordings. Once the transcription of all the interviews was completed, the lead researcher (MR) read it while listening to the recording and corrected any spelling or other errors. The transcripts were anonymized so that the participant cannot be identified from anything that is said (e.g., names, places, significant events) [19, 22, 23, 25]. The three transcriptions in the Arabic language were translated into English by two native speakers. Once all the interviews have been transcribed and checked, they were coded according to the themes and sub-themes and analyzed using thematic analysis, which is a common approach used to analyze qualitative data [18, 21]. Field notes were compiled during an interview, which was a useful complementary source of information to facilitate the whole process, as the gap in time between an interview and the transcribing, and coding could result in memory bias that may affect the interpretation of data [18, 21, 23, 26]. The themes were coded in a way that facilitates retrieval. A qualitative data analysis package QSR NVIVO 12 Pro, was used to organize the data into broad codes [27]. This is carried out by priority issues and questions derived from the aims and objectives of the study as well as issues raised by the respondents.

## Results

Five central themes emerged from the data set (Table 3) followed by a set of sub-themes. The research team, at this point, analyzed all the themes and identified patterns of similarities or differences between participants [20, 21, 25]. Based on the initial coding, the researcher also goes beyond the self-understanding of the interviewee to understand, for example, which factors characterize or drive and influence the perspective of the interviewee [25, 26].

**Table 3:** Themes identified in qualitative interview data

Themes	Explanation
Role and practices	The current roles and practices of community pharmacies
Patient-centered care	What healthcare services would be beneficial for Saudi society
Challenges and drivers of change	What are the barriers of successful transition and how to overcome those barriers
Motivators of change	What motivates the community pharmacy practice towards a successful change
Facilitators of change	What factors helps in successful transition of practice change

### Theme 1: Roles and practices of community pharmacy

#### Current practices in the community pharmacy setting

The majority of participants agreed that the current community pharmacy practice in the KSA varies according to the type and location of the pharmacy. However, participants acknowledged that in KSA, pharmacists are primarily limited to selling and dispensing medicines. When one Ministry of Health official was asked about his opinion on the current practice of community pharmacy, he replied:

They dispense the medication and just make sure the patient knows the medication and they just send the patient home and this is the current practice. OK, now, we have recently seen some new initiatives about pharmaceutical care, but still limited to some chain pharmacies. (MHO-2)

A participant with an academic/research background depicted the current practice as more business-oriented than patient-centered:

The most part of the community pharmacy practice is product oriented and sale oriented rather than actual pharmaceutical care practices or services in the community. (AR-2)

However, participants belonging to the community pharmacies and some of the Ministry of Health officials gave a more optimistic look of the current practices:

The present purpose of community pharmacies is to provide products of quantity and quality that serves the people and they are doing a great service. (MHO-1)

The concept of community pharmacy is considered new in Saudi Arabia but when we talk about it in developed countries, it’s old and it’s applied from long time and practiced from long time, today new and upcoming regulations give more opportunities for patient-centered care facilities. (CP-2)

#### Standards of practice

According to the majority of the study participants, the current rules and regulations of MoH need to be updated to define more transparent practices of community pharmacies in providing patient-centered care. When one practicing academician and researcher was asked about the current standards of practice, he replied:

I think the definition of the policy is the key here. We do have the general regulation of the practice of the pharmacy which includes to some extent the pure legal requirement of things like practice of community pharmacists, requirement of prescriptions for dispensing PoM (Prescription only Medications) etc. However, there needs to be more details about what a pharmacist can do, what kind of services and how they should provide them, title, level, details and depth of documentations and other details. We do have general rules and regulations but they are not detailed enough for the scope of practice. (AR-1)

Contrary to that, when one of the high-ranking officials of the Ministry of Health was asked to give his opinion about the current laws regarding community pharmacy practice, he replied:

The law is there and its clear, and everyone can understand the execution of it. It is based on people's understanding, and there is the understanding gap between the original body which is the Ministry of Health and the local health agencies (called “Muderia” in KSA). I know in the pharmacy, there is no regulation or policy of preventing or violating any pharmacies for opening any private patient-centered care services (MHO-6)

#### Role of community pharmacist

The study participants expressed their opinion that the majority of the community pharmacists are mostly non-Saudis with different practice background and experience:

Most of the pharmacists in the community pharmacies are expatriates and came from Middle Eastern or Asian countries and they bring the practice of community pharmacy in their country to Saudi Arabia. (AR-1)

The participants also shared the opinion that the community pharmacists are not playing the role of providers of patient education effectively as illustrated in the following quotes:

Most of the pharmacists in the community pharmacy, they have a limited knowledge, they do not have a clinical base. (CP-2)

The type of community pharmacists that are right now existing, most of them are expatriates and with all due respect, they are very knowledgeable about the medications but they don’t have the skills to actually provide patient care. (AR-2)

### Theme 2: Patient-centered care

#### Services to support overall healthcare

The stakeholders agreed that there is a dire need that community pharmacies play their role in imporving health outcomes in chronic conditions. Upon asking stakeholders what patient-centered care services would benefit Saudi health consumers, replies included:

Saudi healthcare consumers need preventive services and public education about the chronic diseases, this includes lifestyle modifications to reduce the incidence of the chronic diseases like diabetes and hypertension. (AR-3)

Pharmacy education reconciliation and counselling, these are the two main things that we need to have immediately as top priority followed by more specialized services like providing anti coagulation monitoring, provide HBA1c testing, asthma medication review and education through asthma clinics, antihypertensive education, hypertension management etc. (AR-1)

They should be talking about contraceptives, talking about all the medications and its effect on the pregnancy. Minor illnesses care etc. (AR-2)

#### Patient preferences in using community pharmacies

The participants expressed their opinion that community pharmacies are easily accessible to healthcare consumers without the need for an appointment or wait in a long queue, as illustrated in the following:

First we need to look why the healthcare consumers are preferring community pharmacies. The main reason is accessibility. Community pharmacies are more easily accessible to patients than primary health care centers. Patients avoid waiting for long period of appointments at hospitals, they don’t want to stay in queues in hospitals, long waiting time, shortage of physicians etc. (MHO-2)

#### Effectiveness of patient-centered care

Some of the chain pharmacies in the KSA have initiated diabetic clinics, chronic disease management clinics, and immunization programs for the healthcare consumers with varying degree of success and patient acceptance as illustrated in the following quote by a study participant:

We have been working on to help decrease the complications of the people with diabetes. Medical services provided by our diabetic center results in 20-40% decrease in complications, and the HbA1c which is recorded by our study team, decrease by 1.5%, which is significant. All of these interventions are done by the pharmacists, who didn’t change any medications, all they did was to educate the patients. (CP-1)

Another participant expressed his opinion that despite free healthcare provided by the primary health care centers across the KSA, there is still a need for the patient-centered care initiatives from the community pharmacy:

In my opinion, actually I prefer that government should start primary health care initiatives from community pharmacies. Pharmacies in hospital and primary care set-up have limited spaces, shortage of supplies, expiry issue and administrative issues. Private community pharmacies provide good solutions to all these problems. (MHO-2)

### Theme 3: Drivers of the change process

#### Challenges of current practice

The participants acknowledged that there are structural, administrative, and societal barriers facing the community pharmacies to attain the role of patient-centered care providers. The majority of the study participants expressed their concern that the scope and standards of patient-centered care services need to be strengthened:

I think first one let’s say about regulations. It’s most important and Ministry of Health should have some kind of clear and transparent regulations, answering need of any owners so that it could be adopted by the owners of the community pharmacy. (HC)

One of the issues highlighted by the pharmacy owner and chief operating officers is the lack of economic benefits. Also, there were financial constraints in providing the patient-centered care services as illustrated in the quote below:

The private health sector has increased financial pressures, especially in terms of increasing expenses and in terms of wages and in terms of lack of profitability from agents and on the other hand, too much competition (CP-5)

Another barrier raised by participants was the lack of automation and the lack of access to patient data for community pharmacists. One interviewee attributed this to the unavailability of access to the patient records from the Ministry of Health and inadequacy of software available:

We are missing a lot of tools that could help us in chronic disease management or the services in general. If I told you for example, give me a software that could help us to monitor adverse drug events or drug-drug interactions, patient leaflets in Arabic. (CP-3)

An issue highlighted by the participants was the unavailability of pharmacy technicians in community pharmacies to support the pharmacist in the administrative work. Hence, it increases the workload of pharmacists, resulting in insufficient time to spend with the patients.

We have the pharmacists doing the technicians job, having the tray and taking out medications by himself and doing everything. (AR-2)

The participants strongly emphasized the need for an improved public image of community pharmacists.

The other barrier is that actually related to the public view of community pharmacy, still we did not promote community pharmacy as a health care institution that provide not only medicine but also provide access to accurate information about medicines. (AR-3)

#### Drivers of change

The participants acknowledged that if community pharmacists are encouraged to engage with health consumers, they can deliver patient-centered care services.

If you told the pharmacist that they need to perform these services and if you give them the right tools, if you give them the right time and education towards a particular service, and if you monitor those people, if you give them the smart objectives and you monitor those smart objectives and you manage those schemes, you can get the desired results. (CP-2)

The participants also acknowledged that the big groups of pharmacies and professional organizations such as the Saudi Patient Safety Centre conduct their training programs to provide basic and continuous professional training to their community pharmacists [28].

For diabetes, pharmacists gets training from the diabetes educators, they got the training from governmental entities and they come to provide training. The pharmacists got the training from these government entities for weight management problems, acne and other skin problems, nutritional problems and of course they also have training about side effects of medications, interactions of the medications as well. (CP-2)

The following quote from a policymaker illustrates that the movement to shift the community pharmacy practice from its traditional role to the providers of patient-centered care has started:

I think there's a huge movement right now, towards community pharmacies, I know, these movements come from independent agencies not related to the field itself, like from universities, from research centers, these movements will provide for sure a huge impact in the future. What I'm seeing now, many community pharmacies are getting the idea that the current practice is not the right practice, this is not the ideal practice. So let's take it a step ahead and then provide patient-centered care practices. (MHO-6)

### Theme 4: Motivators of the change process

#### Public healthcare

The participants expressed that monitoring of therapeutic outcomes of the chronic diseases and promoting medication adherence can be the motivation to change practice change at community pharmacies.

You know, when you transfer a discharged patient from hospital, you will lose them most of the time and nobody will follow up with. The patient will be on their own and nobody helps them. If you can have a community pharmacies bridge this gap and then the patients can see a community pharmacists going over their medication’s adherence at different or specific time periods, it’s going to be a huge, huge impact on healthcare consumers. (MHO-6)

#### Business orientation

The participants acknowledged that providing patient-centered care services attracts more healthcare consumers, who appreciate and maintain their loyalty towards their local community pharmacy.

There is tough and hard competition between the pharmacies these days, so the pharmacies have to provide clinical services free of cost to attract customers. (CP-1)

The patient-centered care services affects the loyalty of the business, the people who use these services, they are usually very loyal to the branch and it affects the reputation of the company. (CP-2)

Furthermore, participants indicated that to sustain the patient-centered care services in community pharmacies, appropriate incentives and reimbursement structures need to be in place.

The pharmacist not going to spend half an hour or the 45 minutes of his time to discuss with the patients regarding certain medications or how to improve their control of the diabetes for instance, if he is not going to be reimbursed for that service. (AR-2)

#### Nationalization of the practice

Saudi pharmacy graduates comprise only 4.2% of the total community pharmacies employment by the private sector [29]. The participants acknowledged that the recruitment of Saudi pharmacists in the community pharmacies would attract healthcare consumers.

I think if Saudi pharmacist works as a community pharmacist, they will know my culture and also they can ask me question according to my situation. (MHO-3)

### Theme 5: Facilitators of the change process

#### Regulations

The participants acknowledged that the Ministry of Health is updating the new regulations regarding patient-centered care services offered through community pharmacy.

The current pharmacy regulations are being revised and hopefully will have more detail about the roles and practices of community pharmacists and different patient-centered care services that can be provided through community pharmacies. (AR-1)

However, participants also showed concern that for any updated regulations, local health councils throughout the KSA need to be trained appropriately to enforce these regulations:

If you have the legislation that has been created by a minister from the headquarter and at the time it will be released, all the ministry of health directorate need to be aware and trained of and about how we can inspect and also push for the service or even audit against these centers (CP-2)

#### Collaboration of stakeholders

The majority of stakeholders agreed that the effective collaboration between the Ministry of Health, professional bodies like the Saudi Food and Drug Authority (SFDA) and Saudi Patient Safety Center (SPSC), Saudi Pharmaceutical Society (SPS), community pharmacy owners/CEOs, and Pharmacy Colleges and Research Institutions is necessary for the successful transition of community pharmacy practice in the KSA [28, 30, 31].

The community pharmacy practice can be transformed to a more modern practice and to be successful I believe we need all stake holders in this aspect working together including community pharmacy private sector itself with the Ministry of Health and also with Saudi FDA. (AR-3)

Collaboration is an excellent idea. But you need to again enforce it or have an independent agency like recently in Qassim, the Saudi community pharmacy association, this is an independent agency that can provide different training programs and then it should be certified by Saudi commission of health services and commissioned by a Ministry of Health to provide these training programs. (MHO-6)

## Discussion

### The roles and practices of community pharmacists

The study findings document that the patient-centered care is a new concept in community pharmacies of the KSA. However, the stakeholders in this study perceived that the knowledge and skills of community pharmacists are inadequate to provide patient-centered care services effectively. The study participants acknowledge that big community pharmacy groups in KSA do provide training in diabetes education and other patient-centered care programs in some of their selected community pharmacies [32]. Contrary to that, more than 50% of the community pharmacies in the KSA are independently owned and are more business-oriented than oriented towards patient-centered care. Several studies reported that extending the community pharmacist’s role has proven to be beneficial for health care consumers with improvement in the quality of life, optimization of drug therapy, and reduction of long-term healthcare costs [33–35]. Furthermore, community pharmacists in the developed world are now more integrated into early disease detection, screening, and monitoring of diseases, medication counseling, and improving public health [1, 6, 36].

The extracts of the interview showed that although the MoH regulations are clear regarding community pharmacy practice in the KSA, they need to be refined and updated to define the clear role of community pharmacists in providing patient-centered care services [6, 31]. One interviewee emphasized that the current regulations lack enforcement and there is a need to train the local health care councils in the KSA about the details of the regulations, and to make sure that the regulations are appropriate and equally enforced throughout the country. The stakeholders also acknowledged that an overwhelming majority of community pharmacists in the KSA are non-Saudis with varying degrees of knowledge and skills and inadequate clinical training. Therefore, in order to provide effective patient-centered care services and to get health consumer’s acceptance, community pharmacists should be professionally trained to enhance their clinical knowledge and skills [11, 12, 37].

### Patient-centered care

The majority of the stakeholders acknowledged that to support healthcare in the KSA, community pharmacies need to offer specialized services like diabetes and hypertension education and monitoring, medication counseling, anticoagulation monitoring, asthma clinics, women health initiatives, and treatment of minor illnesses. The studies in the KSA showed that Saudi health care consumers appreciate community pharmacies providing patient-centered care and they prefer community pharmacies to primary health care centers and hospitals to discuss their medication and disease-related problems because of easy accessibility and flexible opening timings [9, 38, 39].

The KSA is a high-income country and its healthcare sector is transforming with the rapid advancement of technology, research, and development [1, 2, 16]. In the developed world, patient-centered care offered through community pharmacies has proven to be beneficial to health care consumers [34, 40]. However, the government and professional bodies in the KSA are grappling with several challenges to achieve the health transformation plan of the vision 2030 [4, 14]. With the growing middle-income population in the KSA, the emergence of lifestyle diseases and illnesses associated with modern and urban lifestyle need to be treated by the health care system [2, 5, 11, 14, 16, 41]. The core findings from the extracts of interviews in this study support the idea of community pharmacists moving beyond the traditional role of selling and dispensing medications and become an effective provider of patient-centered care services.

### Drivers of the change process

The majority of stakeholders in this study mentioned that the MoH policies and regulations should include more explicit role of community pharmacists and the type of services offered, and that can be the main driver of the change towards making community pharmacies as health care providers in the KSA. The participants also highlighted that any future change in policies should take into consideration the business and profitability of community pharmacies.

The participants also identified several challenges of the successful transition of community pharmacies. The importance of an adequate pharmacy dispensing software in the Arabic language, linking of the patient data from primary health care centers and hospitals to the community pharmacies, and overburdening of administrative work are major technical and administrative barriers highlighted by stakeholders [11]. A concern that pharmacy technicians are better utilized was identified by stakeholders.

The stakeholders agreed that professional pharmacy organizations, academics, and researchers can lead and influence the drive for future practice change in community pharmacies. Awareness of the change in the public and raising the competency of current pharmacists through professional development initiates are also important factors highlighted in the interview extracts.

### Motivators of the change process

Although there was awareness among study participants about certain factors that can drive the change process, these were not necessary to motivate present and future pharmacy staff to become actively involved and start the change process. The excerpts from the study participant’s interviews suggest that pharmacists need professional satisfaction through incentives and rewards for their services [11]. The participants in this study also outlined several factors, such as growing competition between pharmacies, nationalization of the practice, and loyalty of health consumers as the motivators of change. These changes can enable community pharmacies to go ahead and implement patient-centered care services.

### Facilitators of the change

Facilitators identified from the interview extracts include remuneration, task delegation, leadership, reorganization, support from governmental entities, and collaboration. The stakeholders described that community pharmacy owners can increase the level of competency of their pharmacists by enrolling them in professional development and certification programs offered by different government entities, which are facilitating the process of change. In addition to supporting from government entities, the value of collaboration between professional bodies, academics, and community pharmacies was highlighted in the interviews through the sub-themes of effective leadership and task delegation. The extracts from the qualitative data suggest that even with appropriate training, collaboration, support, and remuneration, the sustainability of the change process in community pharmacy is at risk if community pharmacists do not move away from their traditional roles and activities to deliver patient-centered care services.

There are some limitations associated with this study. For example, the investigators sought a variety of opinions from key stakeholders belonging to different organizations; the findings of this study may not reflect the views and experiences of all stakeholders. Another limitation of the study is that views from healthcare consumers were also not sought.

## Conclusion

The findings of this exploratory study describe the need for a change in practice in both chain and independent community pharmacies in the KSA. This study identified the drivers, motivators, and facilitators of the change of practice in community pharmacy towards patient-centered care. Defining broader roles of community pharmacists in the regulations set by the Ministry of Health and the utilization of trained pharmacy technicians to support the community pharmacists were identified as important drivers. The nationalization of the practice and incentives and rewards for providing patient-centered care is also considered to be important motivators for practice change in community pharmacy. This study also highlighted the importance of effective collaboration between community pharmacy owners or chief operating officers, professional pharmacy organizations, and pharmacy colleges for the successful implementation of the change process in community pharmacy practice in the KSA.

The motivation of community pharmacists appears to be an important factor for the success of the change process. We hope that the qualitative data obtained in this study will form an essential component of any future investigations related to the community pharmacy practice using quantitative methods.

## Data Availability

The datasets used and/or analyzed during the current study are available from the corresponding author on reasonable request.

## Abbreviations

KSA: The Kingdom of Saudi Arabia
MoH: The Ministry of Health of Saudi Arabia
